# A Meta-Analysis of the Association between *ESR1* Genetic Variants and the Risk of Breast Cancer

**DOI:** 10.1371/journal.pone.0153314

**Published:** 2016-04-12

**Authors:** Taishun Li, Jun Zhao, Jiaying Yang, Xu Ma, Qiaoyun Dai, Hao Huang, Lina Wang, Pei Liu

**Affiliations:** 1 Department of Epidemiology and Biostatistics, School of Public Health, Southeast University, Nanjing, China; 2 National Research Institute for Family Planning, Beijing, China; University of North Carolina School of Medicine, UNITED STATES

## Abstract

**Background:**

Single nucleotide polymorphisms (SNPs) in the estrogen receptor gene (*ESR1*) play critical roles in breast cancer (BC) susceptibility. Genome-wide association studies have reported that SNPs in *ESR1* are associated with BC susceptibility; however, the results of recent studies have been inconsistent. Therefore, we performed this meta-analysis to obtain more accurate and credible results.

**Methods:**

We pooled published literature from PubMed, EMBASE, and Web of Science and calculated odds ratios (ORs) with 95% confidence intervals (CIs) to assess the strength of associations using fixed effects models and random effects models. Twenty relevant case-control and cohort studies of the 3 related SNPs were identified.

**Results:**

Three SNPs of the *ESR1 g*ene, rs2077647:T>C, rs2228480:G>A and rs3798577:T>C, were not associated with increased BC risk in our overall meta-analysis. Stratified analysis by ethnicity showed that in Caucasians, the rs2228480 AA genotype was associated with a 26% decreased risk of BC compared with the GG genotype (OR = 0.740, 95% CI: 0.555–0.987). The C allele of the rs3798577:T>C variant was associated with decreased BC risk in Asians (OR = 0.828, 95% CI: 0.730–0.939), while Caucasians with this allele were found to experience significantly increased BC risk (OR = 1.551, 95% CI: 1.037–2.321). A non-significant association between rs2077647 and BC risk was identified in all of the evaluated ethnic populations.

**Conclusion:**

Rs3798577 was associated with an increased risk of BC in Caucasian populations but a decreased risk in Asians. Rs2228480 had a large protective effect in Caucasians, while rs2077647 was not associated with BC risk.

## Introduction

Breast cancer (BC) is the most common cancer and is a major cause of death in women worldwide [1]. Previous evidence has suggested that genetic variants and environmental factors may contribute to the development of BC [2–5]. Additionally, estrogen plays a well-known crucial role in the pathogenesis and progression of BC [6]. Estrogen stimulates breast epithelial cell growth, primarily by binding to the estrogen receptor (ER), which increases cancer risk [7]. The ER has two major forms, alpha and beta, both of which can be expressed in normal and neoplastic breast tissue. ER-alpha (ER-**α**), encoded by the *ESR1* gene, is associated with BC risk because it acts as a transcriptional regulator by interacting with estrogen and other coactivator proteins.

The human *ESR1* gene is a steroid hormone receptor gene located on chromosome 6 at 6q25.1. It contains eight exons spanning ~295 kb [8]. Many SNPs in *ESR1* gene were shown to be associated with BC risk, including rs2234693, rs1801132, rs9340799, rs2077647, rs2228480 and rs3798577, and also, studies have showed that the genetic variants played important roles in the transcription and protein expression[9, 10]. Recently, several Meta-analysis showed that genetic variants at rs2234693, rs1801132 and rs9340799 loci were associated with the increased risk of BC[11–14], while the effects of SNPs in rs2077647, rs2228480 and rs3798577 were also in controversy. Several studies evaluated these three SNPs and their association with BC [15–34]. This review focuses on variants discovered through candidate gene studies and not genome-wide association studies (GWAS). For the three SNPs 20 eligible studies were included in our work, every single SNPs included 11 eligible studies. Two of these studies reported positive effects of rs2228480 on BC risk, while the other studies observed no association between the rs2228480 *ESR1* genetic variant and BC risk. One study showed a protective effect of rs2077647 on BC risk, another study reported that *ESR1* rs2077647 increased BC risk, and the remaining studies failed to replicate these associations. Three studies showed that the rs3978577 SNP, which is located in the 3’ UTR of ER-α, increased the overall risk of BC, one study provided evidence that it decreased BC risk, and the others also failed to replicate these associations.

Although rs3798577 and rs2228480 were discussed in a meta-analysis in 2010, the analysis included only 4 studies for each SNP [12]. However, the number of studies included in a meta-analysis directly influences the credibility and stability of the findings. The time of analysis is also a key factor for meta-analyses, and several new studies, which could change the results of the meta-analysis, have been conducted in the 5 years since 2010. Therefore, to more accurately assess the relationships between these three *ESR1* polymorphisms and the risk of BC, a new meta-analysis that integrated more recent studies with earlier publications was conducted.

## Materials and Methods

### Publication search

Relevant English papers published before October 1, 2015, were identified through a search of the PubMed, Web of Science, EBSCO and EMBASE databases using the following terms: (“genetic polymorphism” or “single nucleotide polymorphism” or “SNP” or “gene mutation”) and (“breast cancer” or “breast neoplasm” or “carcinogenesis” or “breast carcinoma” or “breast tumor” or “BC” or “mammary cancer”) and (“*ESR1*” or “Estrogen receptor α” or “ER alpha” or “Estrogen receptor alpha” or “ERα”). Google Scholar was also used to search for relevant studies. Chinese papers were selected by searching the WanFang Data, Chongqing VIP (CQVIP), and China National Knowledge Infrastructure (CNKI) databases using the same search terms. The references of eligible articles were also inspected to find other potential studies. Only studies published in English or Chinese were included in this meta-analysis; any disagreement was resolved via discussion between two of the authors (H.H. and J.Z.). E-mail was used to contact study authors to obtain full text articles or missing data. This study was performed in accordance with the PRISMA statement checklist (S1 PRISMA Checklist) and the Meta-analysis of Genetic Association Studies checklist (S2 Checklist). The full details of the database searches used to identify the studies included in this meta-analysis have been provided in the supplementary materials (S1 Text).

### Inclusion of relevant studies

The inclusion criteria were the following: (1) case-control or cohort study focused on associations between *ESR1* gene polymorphisms and BC susceptibility; (2) availability of odds ratios (ORs) with 95% confidence intervals for polymorphisms and haplotypes or sufficient genotyping data to estimate these parameters; and (3) all diagnoses of BC confirmed by pathological or histological examination. Reviews, simple commentaries, case reports and meta-analyses were excluded. For overlapping studies, only the study with the largest sample was included.

### Data extraction and quality assessment

The data from the published studies were extracted independently by two of the authors, and consensus was reached on all of the items. For each study, the following variables were collected: first author’s name or study organization name, year of publication, area, language, ethnicity, study methods, number of cases and controls, sources of cases and controls, allele and genotype frequencies, Hardy-Weinberg equilibrium (HWE), OR value, statistical power and minor allele frequency (MAF) in the controls. OR adjustment factors are not listed in our tables because every study used different factors for OR adjustment; therefore, it was difficult to find common factors for our meta-analysis.

The Newcastle-Ottawa Quality Assessment Scale (NOS) (S2 Text) was used independently by two authors (T.S.L. and J.Y.Y.) to evaluate the quality of the included studies (http://www.ohri.ca/programs/clinical_epidemiology/oxford.asp). The NOS contains two different quality assessment scales for case-control studies and cohort studies. The two different forms each consist of three groupings, but the grouping items differ. The NOS identifies “high”-quality choices with a “star”, with a maximum of one “star” for each item within the “Selection” and “Exposure/Outcome” categories, and a maximum of two “stars” for “Comparability”. To obtain objective outcomes, any disagreement was discussed, and another author was consulted.

### Statistical analysis

The association of the *ESR1* polymorphisms with BC susceptibility was measured by ORs with 95% CIs in four genetic models, including a variant heterozygote versus wild-type homozygote model, a variant homozygote versus wild-type homozygote model, a dominant model, and a recessive model. Between-study heterogeneities were estimated using the *χ*^*2*^-based Q test [35], and the heterogeneity was considered significant at *P*<0.05. The *I*^*2*^ statistic was then used to quantitatively evaluate heterogeneity (*I*^*2*^<25%, low heterogeneity; 25%≤*I*^*2*^≤75%, moderate heterogeneity; *I*^*2*^>75%, high heterogeneity) [36]. When a significant Q test result (*P*<0.05) or *I*^*2*^>50% indicated heterogeneity among the studies, a random effects model (DerSimonian Laird method) was used to conduct the meta-analysis; otherwise, a fixed effects model (Mantel-Haenszel method) was used. To explore the sources of cross-study heterogeneity, subgroup analysis by ethnicity was performed. HWE of the genotype frequencies in the control group was assessed by the goodness-of-fit *χ*^*2*^ test. Sensitivity was evaluated by omitting each study one at a time to assess the influence of each study on the overall estimate [37]. Publication bias was assessed using funnel plots and Egger’s tests [38, 39]. The fail-safe number (N_fs_) was also used to assess the stability of the results through comparison with the number of relevant included studies. All of the *P* values were two sided, with significance defined at 0.05. All analyses were performed using Review Manager software (version 5.0; Oxford, United Kingdom). The gene data for the heterogeneity analysis were download from the International HapMap Project (http://hapmap.ncbi.nlm.nih.gov/). Allele frequencies for the three polymorphisms in different populations were assessed by the goodness-of-fit *χ*^*2*^ test, and the linkage disequilibrium (LD) analysis was performed using Haploview software (version 4.0).

## Results

### Study selection and characteristics

**Fig 1:**
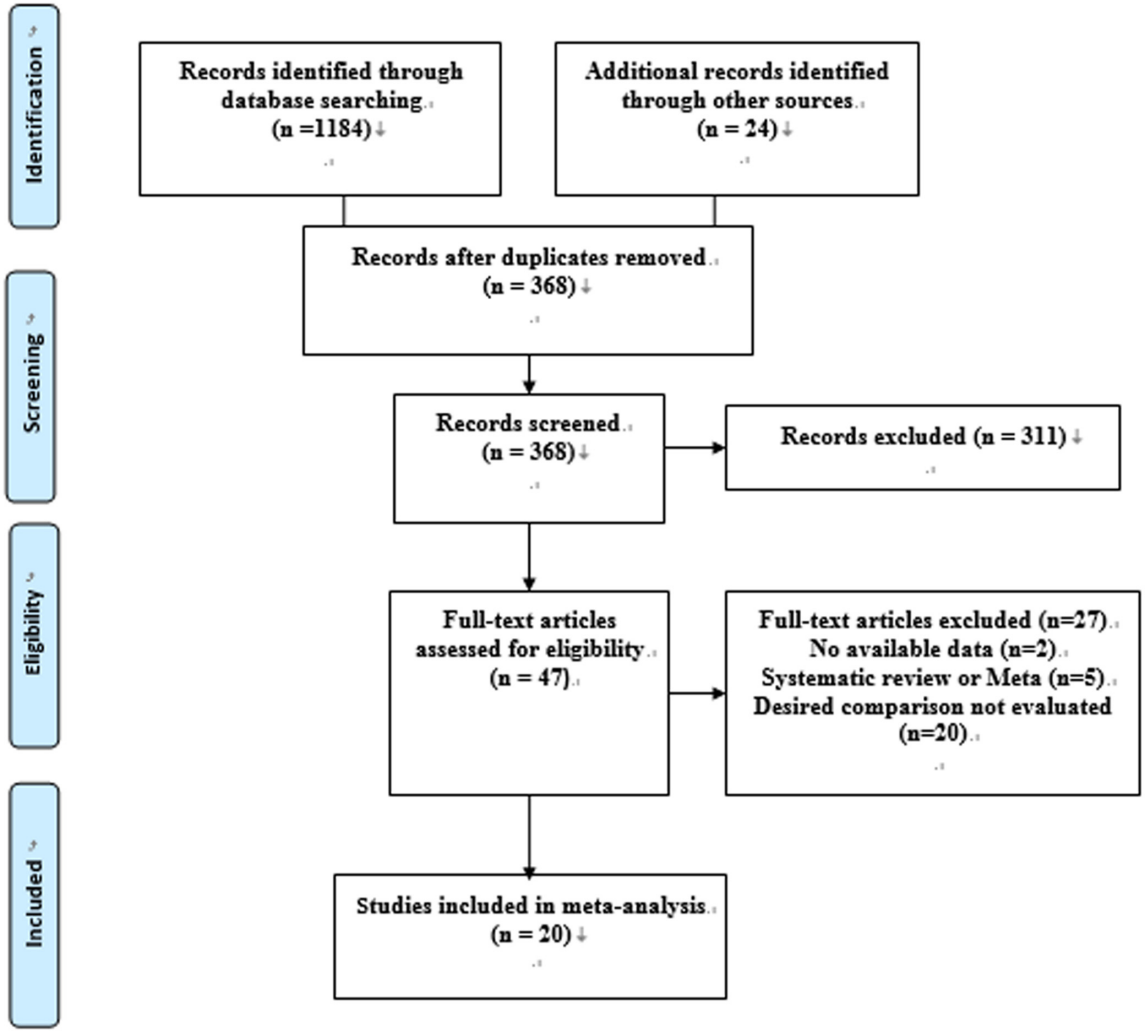
Flowchart of the selection of studies included in the meta-analysis.

The initial search of EMBASE, PubMed, and Web of Science yielded 1184 relevant articles, and an additional 24 records were identified through other sources. Following the deletion of duplicate results obtained from multiple databases, 368 records remained. After the titles and abstracts of the 368 articles were reviewed, 47 full-text articles were finally considered eligible. Ultimately, 20 eligible studies [15–34] were included in our analysis. The excluded full-text articles are listed in the supplementary material (S1 Table). The study selection process is presented in detail in Fig 1.

The characteristics of the 20 eligible studies are presented in Table 1. Only two studies [30, 34] published in Chinese were included in this meta-analysis; some studies [22, 23, 25, 31, 33] did not provide information about genotypes. The factors for OR adjustment were primarily age, family history of BC, and age at first full-term pregnancy. Other basic information, including the first author’s name, year of publishing, study area, ethnicity of the study population, study methods, number of cases and controls, and source of cases and controls, are listed in Table 1. All of the studies indicated that the distribution of genotypes in the controls was consistent with HWE except for two studies of rs2077647 [28, 30]. Only five studies achieved statistical power greater than 80% [16, 17, 24, 29, 33]. The supplementary information includes the results of the NOS-based quality assessment of the 20 studies (S2 Table), a detailed summary of the genotype and allele frequencies (S3 Table), detailed information about the three SNPs in the four different models (S4, S5 and S6 Tables), and some additional characteristics of all of the eligible studies (S7 Table).

**Table 1:** Characteristics of all of the eligible studies of the *ESR1* polymorphisms and breast cancer.

SNP	Author	Year	Country/ Area	Ethnicity	Sample size		HWE	MAF	Study Method	Case		Control		OR(95%CI)
					case	control				A	a	A	a	
rs2228480										G	A	G	A	
	Jeon, S.[15]	2010	Korea	Asian	864	723	0.584	0.185	CC	1248	300	1100	250	1.40(0.81–2.52)
	Anghel, A.[16]	2010	Romania	Caucasian	103	92	0.596	0.137	CC	163	43	145	23	1.01(0.06–16.6)
	Yu, Jyh-Cherng[17]	2006	Taiwan	Asian	468	470	0.467	0.228	CC	702	232	723	213	1.27(0.95–1.70)
	Wang, Y. R.[18]	2014	China	Asian	1064	1073	0.295	0.227	CC	1704	420	1653	485	0.84(0.72–0.98)
	Gallicchio, L.[21]	2006	USA	Caucasian	91	1347	0.702	0.177	Cohort	136	24	2040	440	1.42(0.34–6.01)
	Hsiao, W. C.[19]	2004	Taiwan	Asian	189	177	0.628	0.184	CC	308	70	289	65	-
	Bosviel, Rémy[20]	2012	France	Caucasian	902	990	0.094	0.178	CC	1496	306	1617	351	-
	Tapper, William^a^[22]	2008	UK	Caucasian	899	2980	0.987	0.231	Cohort	1437	361	4584	1376	0.84(0.73–0.95)
	Wang, J.^a^[23]	2013	China	Asian	206	230	0.995	0.175	CC	331	81	378	80	1.15(0.82–1.63)
	Kallel, Imen[24]	2009	Tunisia	African	142	240	0.103	0.229	CC	236	46	370	110	2.33(0.83–6.53)
	Son, B. H.^a^[25]	2014	Korea	Asian	830	390	0.360	0.233	CC	1398	326	598	182	0.81(0.62–1.06)
rs2077647										T	C	T	C	
	Fernandez, L. P.[26]	2006	Spanish	Caucasian	550	564	0.441	0.477	CC	606	464	564	514	0.74(0.53–1.02)
	Nyante, Sarah J.[27]	2015	USA	Mixed	1972	1766	0.190	0.483	CC	2054	1890	1835	1711	0.99(0.81–1.20)
	Anghel, A.[16]	2010	Romania	Caucasian	103	92	0.584	0.349	CC	130	76	108	58	1.16(0.43–3.09)
	Gallicchio, L.[21]	2006	USA	Caucasian	91	1347	0.917	0.488	Cohort	88	90	1312	1250	1.14(0.65–1.99)
	Hsiao, W. C.[19]	2004	Taiwan	Asian	189	177	0.056	0.404	CC	257	121	211	143	-
	Diergaarde, B.[28]	2008	USA	Caucasian	324	651	0.007	0.506	CC	320	328	643	659	1.00(0.80–1.40)
	Tse[29]	2006	Hongkong	Asian	336	313	0.698	0.413	CC	431	241	366	258	0.58(0.66–0.94)
	Xu, Yingchun^b^[30]	2004	China	Asian	193	132	0.000	0.636	CC	252	134	96	168	-
	Wang, J.^a^[23]	2013	China	Asian	206	230	0.960	0.428	CC	237	175	263	197	0.99(0.76–1.30)
	O'Brien, K. M.^a^[31]	2014	USA	Mixed	1260	1817	0.995	0.490	CC	1260	1260	1854	1780	-
	Son, B. H.^a^[25]	2014	Korea	Asian	830	390	0.224	0.336	CC	1028	632	518	262	1.37(1.05–1.79)
rs3798577										T	C	T	C	
	Zhang, L.[32]	2009	China	Asian	300	390	0.287	0.455	CC	359	241	425	355	1.37(0.84–2.23)
	Nyante, Sarah J.[27]	2015	USA	Mixed	1972	1766	0.123	0.464	CC	2131	1811	1905	1647	0.94(0.78–1.14)
	Wang, Y. R.[18]	2014	China	Asian	1064	1073	0.627	0.463	CC	1199	919	1151	993	0.90(0.79–1.02)
	Fernandez, L. P.[40]	2006	Spanish	Caucasian	550	564	0.292	0.454	CC	570	488	597	497	1.04(0.75–1.46)
	Anghel, A.[16]	2010	Romania	Caucasian	103	92	0.561	0.433	CC	75	131	101	77	7.50(2.86–19.65)
	Tapper, William^a^[22]	2008	UK	Caucasian	899	2980	0.997	0.471	Cohort	902	896	3151	2809	1.11(1.00–1.24)
	Wang, J.^a^[23]	2013	China	Asian	206	230	0.948	0.413	CC	249	163	270	190	0.93(0.71–1.22)
	SD Boone^a^[33]	2013	USA	Caucasian	683	705	0.989	0.426	CC	711	655	809	601	1.36(1.04–1.76)
	O'Brien, K. M.^a^[31]	2014	USA	Mixed	1260	1817	0.993	0.470	CC	1311	1209	1927	1707	-
	Zhang, Lina^b^[34]	2008	China	Asian	300	390	0.287	0.455	CC	359	241	425	355	-
	Son, B. H.^a^[25]	2014	Korea	Asian	830	390	0.149	0.423	CC	1059	601	450	330	0.76(0.58–1.00)

*HWE* Hardy-Weinberg equilibrium, *MAF* minor allele frequency, *A* major allele, *a* minor allele, *OR* odds ratio, *CI* confidence interval, *CC* case control study

^a^ Genotype frequency data were not supplied and were calculated based on raw data

^b^ Study published in Chinese

### Overall meta-analysis and stratified analyses

The evaluation of the associations of these three polymorphisms with BC risk and the stratified analyses by ethnicity are presented in Table 2.

**Table 2:** Pooled ORs of the three SNPS in the different genetic models and in different ethnic subgroups.

SNP	Ethnicity	Comparisons	Case/Control	AB vs. AA		BB vs. AA		(BB+AB) vs. AA		BB vs. (AB+AA)	
				OR(95%CI)	*P*^a^	OR(95%CI)	*P*^a^	OR(95%CI)	*P*^a^	OR(95%CI)	*P*^a^
rs2228480	Asian	6	3621/3063	0.99(0.84–1.17)	0.903	0.96(0.75–1.23)	0.759	1.00(0.84–1.20)	0.980	0.96(0.75–1.23)	0.751
	Caucasian	4	1995/5409	0.96(0.76–1.22)	0.754	***0*.*74(0*.*55–0*.*99)***	0.040	0.94(0.75–1.19)	0.624	0.77(0.58–1.03)	0.075
	African	1	142/240	0.69(0.43–1.10)	0.121	0.43(0.15–1.20)	0.106	0.64(0.41–1.00)	0.051	0.48(0.17–1.33)	0.157
	Overall	11	5758/8712	0.96(0.84–1.08)	0.471	0.84(0.70–1.00)	0.056	0.95(0.84–1.09)	0.469	0.85(0.71–1.02)	0.090
rs2077647	Asian	5	1754/1241	1.06(0.90–1.24)	0.515	0.57(0.26–1.23)	0.153	1.02(0.87–1.19)	0.824	0.88(0.71–1.09)	0.253
	Caucasian	4	1051/2554	0.87(0.72–1.05)	0.134	0.90(0.72–1.12)	0.348	0.88(0.74–1.04)	0.139	0.98(0.82–1.19)	0.869
	Mixed	2	3232/3590	0.98(0.87–1.10)	0.713	1.02(0.89–1.17)	0.727	0.99(0.89–1.11)	0.894	1.04(0.93–1.16)	0.513
	Overall	11	6037/7385	0.97(0.90–1.06)	0.543	0.79(0.60–1.05)	0.102	0.97(0.90–1.05)	0.512	1.00(0.91–1.09)	0.970
rs3798577	Asian	5	2695/2472	***0*.*83(0*.*73–0*.*94)***	0.019	***0*.*72(0*.*61–0*.*85)***	0.000 ^b^	***0*.*78(0*.*69–0*.*88)***	0.000	***0*.*80(0*.*70–0*.*93)***	0.003
	Caucasian	4	2214/4321	1.32(0.98–1.78)	0.071	***1*.*55(1*.*04–2*.*32)***	0.033 ^b^	***1*.*39(1*.*01–1*.*91)***	0.041	***1*.*21(1*.*00–1*.*47)***	0.050
	Mixed	2	3231/3593	1.05(0.93–1.17)	0.438	1.01(0.88–1.16)	0.875	1.03(0.93–1.15)	0.537	0.98(0.87–1.12)	0.812
	Overall	11	8140/10386	1.00(0.88–1.14)	0.670	1.00(0.81–1.22)	0.971	0.98(0.85–1.15)	0.837	0.98(0.87–1.11)	0.785

*A* major allele, *B* minor allele, AB variant heterozygote, AA wild-type homozygote, BB variant homozygote, AB vs. AA: variant heterozygote versus wild-type homozygote, BB vs. AA: variant homozygote versus wild-type homozygote, (BB+AB) vs. AA: dominant model, BB vs. (AB+AA): recessive model

^a^ Significance tests of ORs

**Fig 2:**
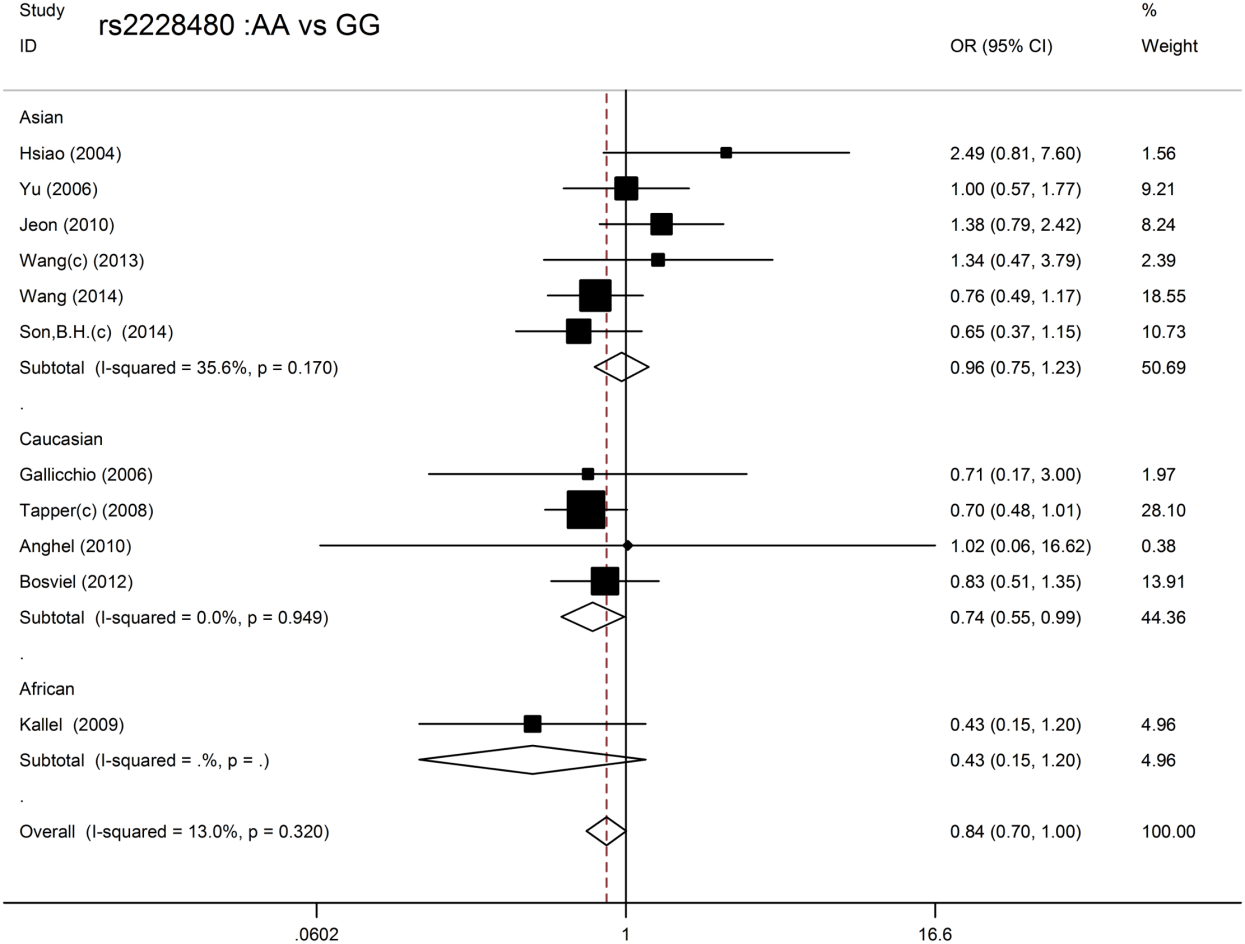
Forest plot of the association between rs2228480 and breast cancer risk in different ethnicities in the variant homozygote versus wild-type homozygote model.

For rs2228480, the eligible studies included 5758 BC patients and 8712 control subjects. The *P* value for heterogeneity was less than 0.05 in the dominant model and variant heterozygote versus wild-type homozygote model; therefore, the ORs were pooled in a random effects model. No significant association was found between the rs2228480 genetic variant and BC in any of the four models, and no significant effect was found in Asians. However, Caucasians carrying the rs2228480 TT genotype had a 26% decreased risk of BC compared with those with the CC genotype (OR = 0.74, 95% CI: 0.55–0.99, *P* = 0.040, N_fs =_ 3) (Table 2) (Fig 2).

**Fig 3:**
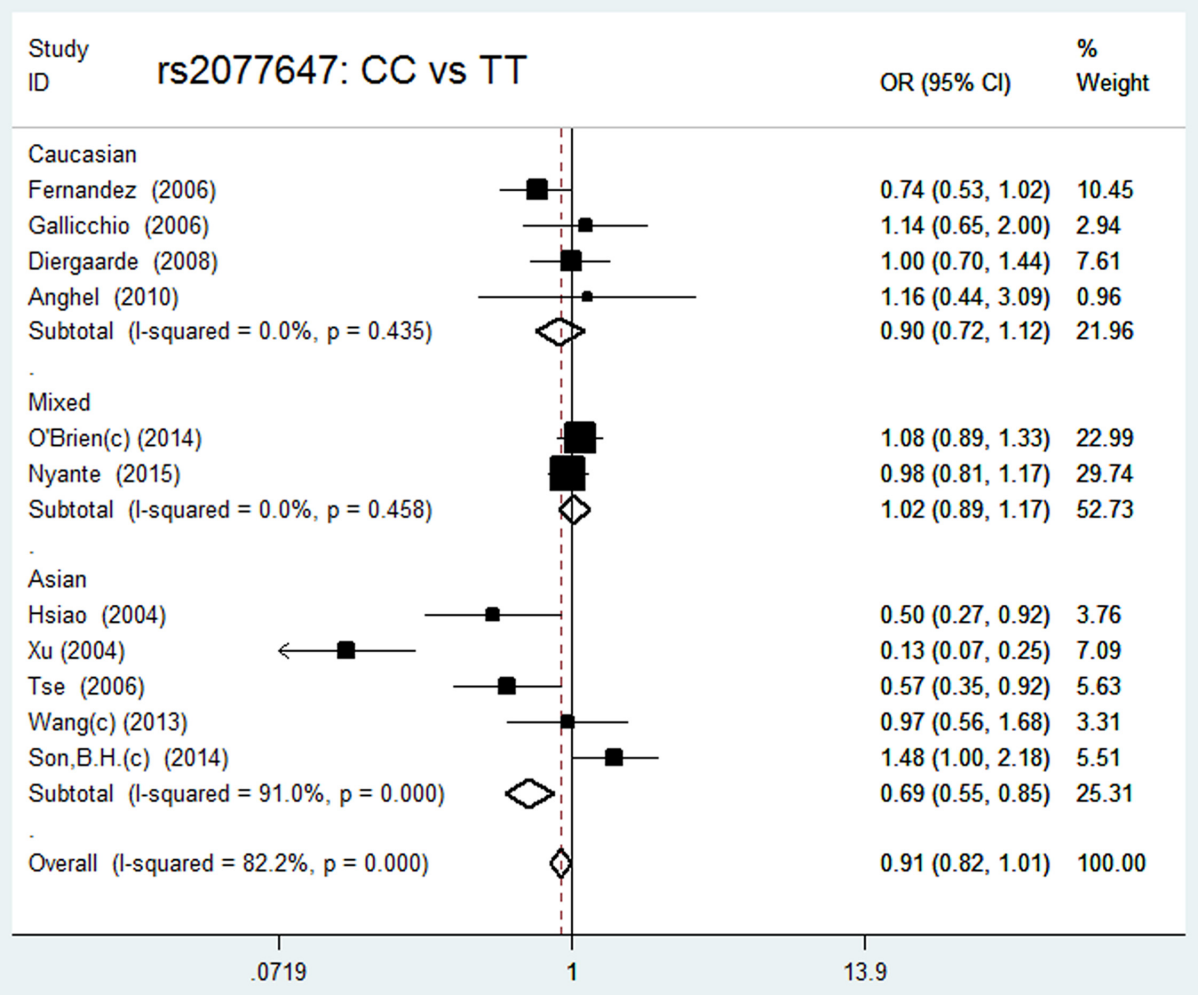
Forest plot of the association between rs2077647 and breast cancer risk in different ethnicities in the variant homozygote versus wild-type homozygote model.

The values in italics indicate *P* values less than <0.05, which were considered to be statistically significant. For rs2077647, the eligible studies included 6037 BC patients and 7385 control subjects. In the overall population, the Q test of heterogeneity was significant in the variant homozygote versus wild-type homozygote model, and the analysis was conducted using random effect models. There was no obvious association between the SNP and BC risk in any of the genetic models. The subgroup analysis revealed similar results in the Asian, Caucasian and mixed ethnic groups (Table 2) (Fig 3).

**Fig 4:**
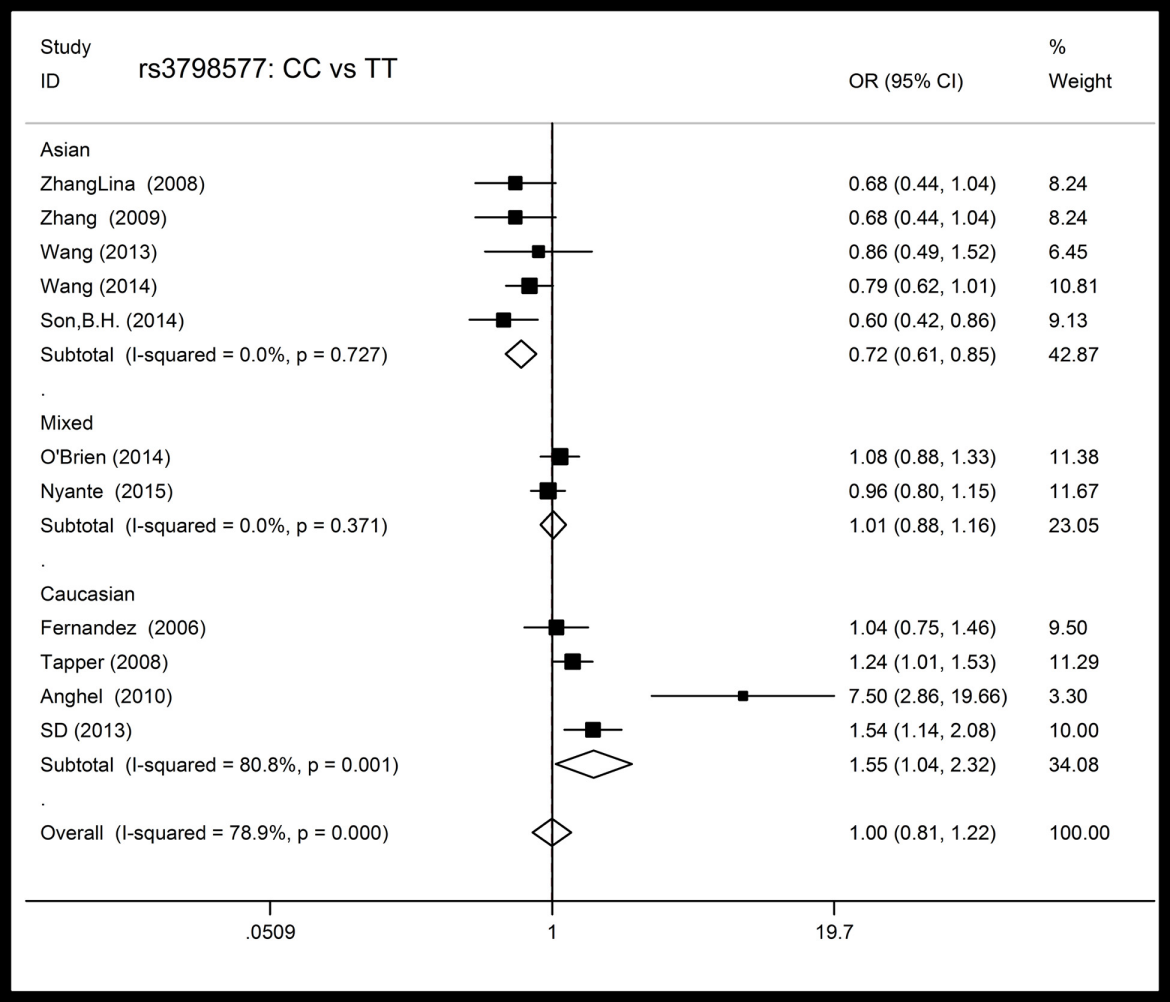
Forest plot of the association between rs3798577 and breast cancer risk in different ethnicities in the variant homozygote versus wild-type homozygote model.

For rs3798577, the eligible studies included 8140 BC patients and 10386 control subjects. In the overall population, there was significant heterogeneity in all of the genetic models, so the analysis was conducted using random effect models. We failed to find a significant main effect on BC risk in any of the test models. In the ethnicity subgroup analysis, we found that among Asians, the variant C allele was associated with a decreased BC risk in all of the genetic models (CT vs. TT: OR = 0.83, 95% CI: 0.73–0.94, *P* = 0.019, N_fs =_ 11; CC vs. TT: OR = 0.72, 95% CI: 0.61–0.85, *P* = 0.000, N_fs =_ 23; (CT+CC) vs. TT: OR = 0.78, 95% CI: 0.69–0.88, *P* = 0.000, N_fs =_ 29; CC vs. (TT+CT): OR = 0.80, 95% CI: 0.70–0.93, *P* = 0.003, N_fs =_ 11). In the dominant, recessive and variant homozygote versus wild-type homozygote models, Caucasians carrying the variant C allele were found to experience significantly increased BC risk (CC vs. TT: OR = 1.55, 95% CI: 1.04–2.32, *P* = 0.033, N_fs =_ 26; (CT + CC) vs. TT: OR = 1.39, 95% CI: 1.01–1.91, *P* = 0.041, N_fs =_ 23; CC vs. (TT+CT): OR = 1.21, 95% CI: 1.00–1.47, *P* = 0.050, N_fs =_ 8). However, no significant associations were found in the mixed population. The data are presented in detail in Table 2 and Fig 4.

### Publication bias

**Fig 5:**
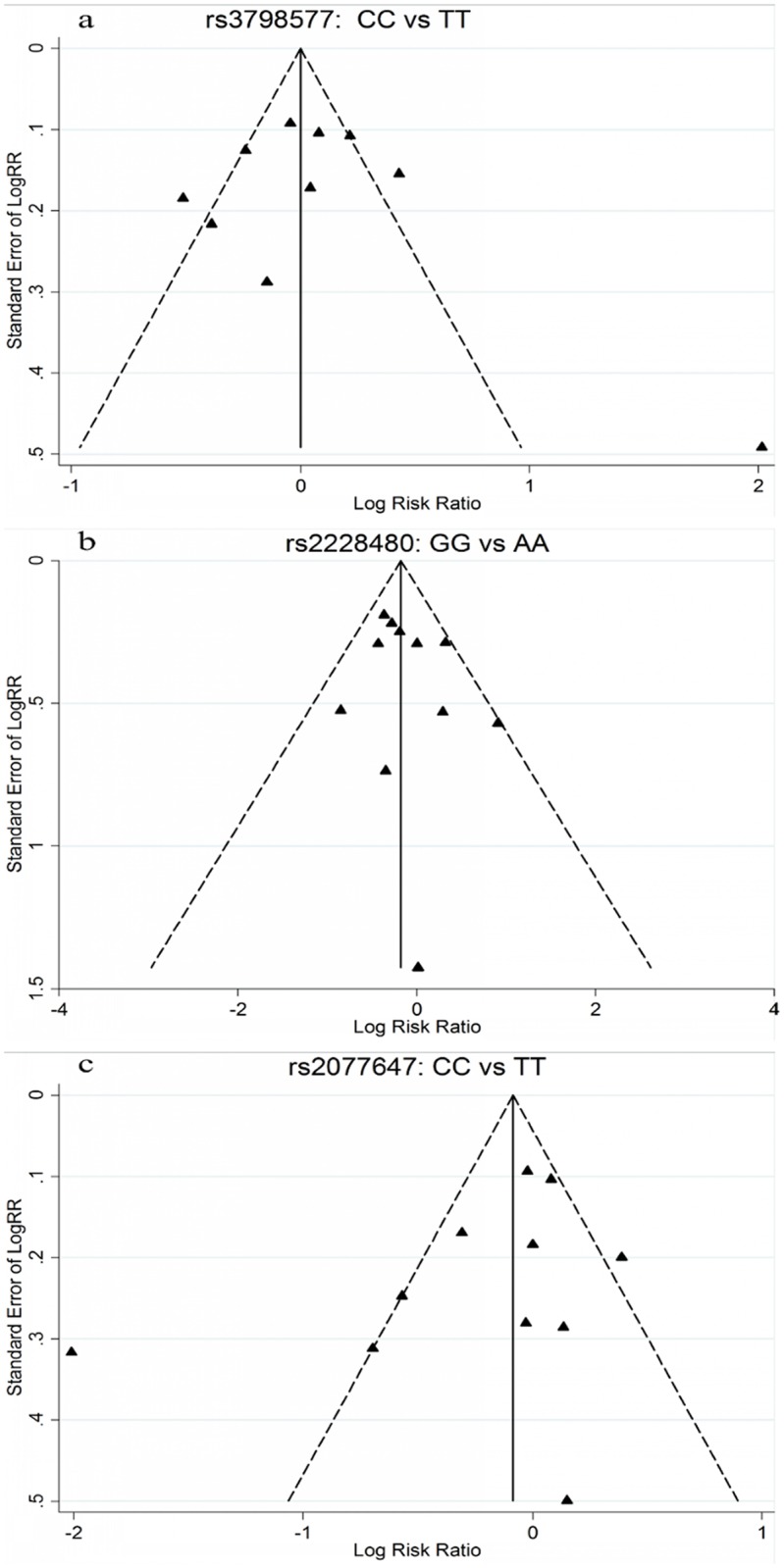
Funnel plot analysis to detect publication bias in the variant homozygote versus wild-type homozygote model. a Funnel plot analysis of rs3798577; b Funnel plot analysis of rs2228480; c Funnel plot analysis of rs2077647.

Funnel plots and Egger’s tests were used to assess the publication bias of the included studies. The funnel plots did not reveal any evidence of obvious asymmetry in the three SNPs in the variant homozygote versus wild-type homozygote model (Fig 5). Egger’s tests (all *P* values for Egger’s test>0.05) also showed that there was no evidence of publication bias for any of the three polymorphisms (t = -0.89, *P* = 0.398 for rs2228480; t = -1.40, *P* = 0.196 for rs2077647; and t = 0.22, *P* = 0.829 for rs3798577).

### Sensitivity analysis

**Fig 6:**
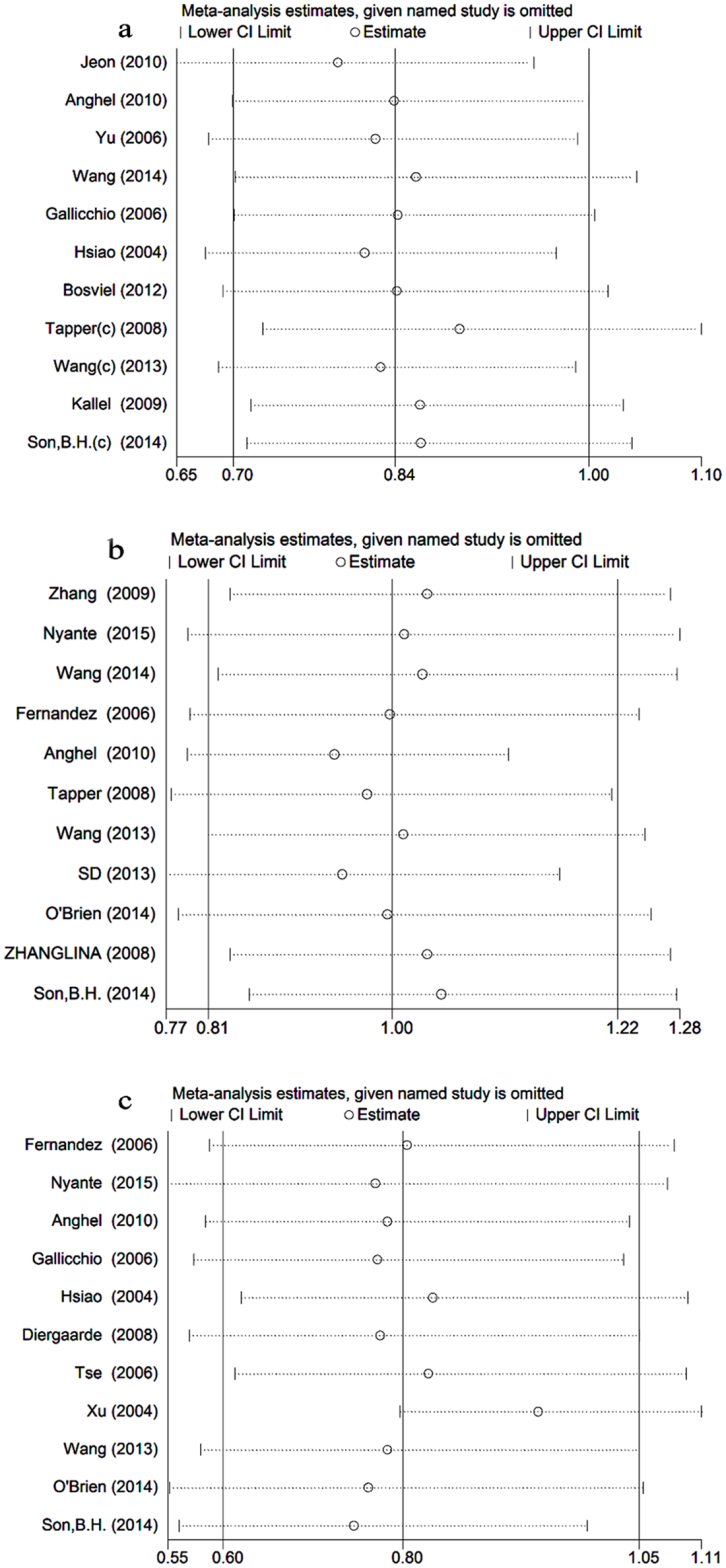
Sensitivity analysis of the meta-analysis of the association of the three *ESR1* gene polymorphisms with breast cancer risk in the variant homozygote versus wild-type homozygote model. a Sensitivity analysis of rs2228480. b Sensitivity analysis of rs3798577. c Sensitivity analysis of rs2077647. The vertical axis indicates the overall OR, and the two vertical axes indicate the 95% CI. Every hollow round indicates the pooled OR when the left study was omitted from the meta-analysis.

Sensitivity analyses were performed to evaluate the effect of each study on the pooled ORs through sequential removal of individual studies (Fig 6). No individual study significantly altered the pooled ORs for any of the three SNPs in the variant homozygote versus wild-type homozygote model, and similar results were also achieved for the other test models. Therefore, the data in this meta-analysis were relatively stable and credible. The N_fs_ of the positive result indicated that the results in this meta-analysis were also relatively stable and credible.

### Heterogeneity analysis

Heterogeneity analyses were performed to explore the reason for the heterogeneity in the associations found in the Caucasian and Asian populations. Measures of LD and allele frequencies for the three polymorphisms in the different populations comprised the two parts of this analysis.

Allele frequencies for the three polymorphisms in the different populations are listed in Table 3. The results (all *P* values for *χ*^*2*^ test >0.05) showed that there was no heterogeneity in the allele frequencies for the three polymorphisms in the different populations (*χ*^*2*^ = 6.971, *P* = 0.073 for rs2077647; *χ*^*2*^ = 0.643, *P* = 0.887 for rs2228480; and *χ*^*2*^ = 2.296, *P* = 0.513 for rs3798577).

**Table 3:** Allele frequencies in different populations for the three polymorphisms.

SNP	Population	Genotype frequencies										Allele frequencies						
												Ref-allele			Other-allele			
		genotype	freq	count	genotype	freq	count	genotype	freq	count	Total	allele	freq	count	allele	freq	count	*P*^a^
rs2077647	CEU	A/A	0.379	22	A/G	0.397	23	G/G	0.224	13	58	A	0.578	67	G	0.422	49	0.073
	CHB	A/A	0.422	19	A/G	0.422	19	G/G	0.156	7	45	A	0.633	57	G	0.367	33	
	JPT	A/A	0.386	17	A/G	0.477	21	G/G	0.136	6	44	A	0.625	55	G	0.375	33	
	YRI	A/A	0.237	14	A/G	0.475	28	G/G	0.288	17	59	A	0.475	56	G	0.525	62	
rs2228480	CEU	G/G	0.733	44	A/G	0.233	14	A/A	0.033	2	60	G	0.850	102	A	0.150	18	0.887
	CHB	G/G	0.733	33	A/G	0.244	11	A/A	0.022	1	45	G	0.856	77	A	0.144	13	
	JPT	G/G	0.756	34	A/G	0.222	10	A/A	0.022	1	45	G	0.867	78	A	0.133	12	
	YRI	G/G	0.783	47	A/G	0.200	12	A/A	0.017	1	60	G	0.883	106	A	0.117	14	
rs3798577	CEU	T/T	0.300	18	C/T	0.450	27	C/C	0.250	15	60	T	0.525	63	C	0.475	57	0.513
	CHB	T/T	0.378	17	C/T	0.444	20	C/C	0.178	8	45	T	0.600	54	C	0.400	36	
	JPT	T/T	0.378	17	C/T	0.489	22	C/C	0.133	6	45	T	0.622	56	C	0.378	34	
	YRI	T/T	0.333	20	C/T	0.500	30	C/C	0.167	10	60	T	0.583	70	C	0.417	50	

Population description:

YRI: Yoruba in Ibadan, Nigeria

JPT: Japanese in Tokyo, Japan

CHB: Han Chinese in Beijing, China

CEU: CEPH (Utah residents with ancestry from northern and western Europe)

^a^: Significance tests of allele frequencies among populations

**Fig 7:**
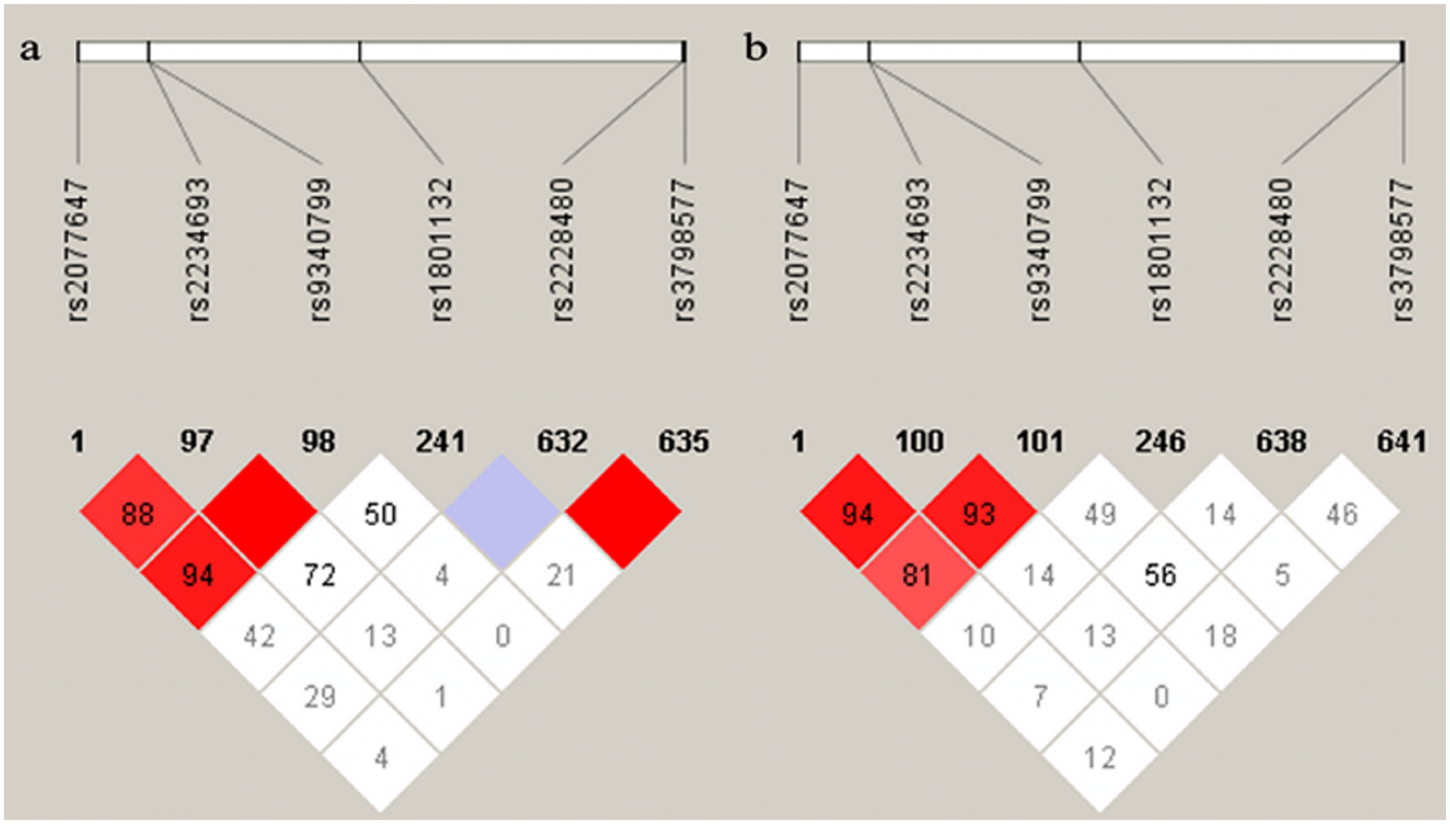
The pattern of linkage disequilibrium in alleles of the *ESR1* gene in the different populations, with their |D’|. a CEU: CEPH (Utah residents with ancestry from northern and western Europe). b CHB+JPT: Han Chinese in Beijing, China and Japanese in Tokyo, Japan.

The LD plots of all SNPs that were previously found to be associated with BC in different populations are presented in Fig 7. The results showed that there was heterogeneity in LD for the three polymorphisms in the different populations. In the Caucasian group, rs2228480 and rs3798577 were found to be in linkage disequilibrium. However, no linkage disequilibrium was found between rs2228480 and rs3798577 in the Asian population. The other SNPs showed the same pattern of linkage disequilibrium between Asian and Caucasian populations. The LD plots for other populations were presented as supporting information (S1 Fig).

## Discussion

Genetic variants in the *ESR1* gene have been shown to alter ER-α expression and to therefore modulate downstream signaling and BC susceptibility [41]. The *ESR1* gene plays an important role in the progression of breast carcinogenesis by inducing cell proliferation, programming cell death and accumulating genetic mutations [42]. Many genetic variants in the *ESR1* gene that are correlated with susceptibility have been identified.

Our findings showed that the SNPs rs2077647, rs2228480 and rs3798577 were not associated with BC risk in the four test models included in our overall meta-analysis. After the data were stratified by ethnicity, the analysis demonstrated that rs3798577 was associated with an increased risk of BC in Caucasians but had a protective effect in Asians. SNP rs2228480 also had a significant association with BC risk in Caucasians. The strength of the association of rs2228480 and rs3798577 with BC risk varied greatly across ethnic groups. An earlier study [13] indicated that the tremendous differences in genetic backgrounds between ethnicities and the different LD patterns among different ethnic populations might contribute to this phenomenon. Comparison of allele frequencies and LD patterns between the different ethnic populations were made to explore possible reasons for the observed interaction.

Comparison of allele frequencies showed that there were not heterogeneous among the different populations, but the LD plots for the rs3798577 in the different populations showed an opposite result. Hence, two potential reasons for the reversed interaction in rs3798577 between the different ethnic populations can be proposed. First, it may be caused by the differences in the function of genetic variants among different ethnic populations. Second, heterogeneity in LD for the rs3798577 in the different populations is also the possible reason.

GWAS have provided a powerful approach for identifying common disease alleles. Recent GWAS have identified several genetic susceptibility loci for BC, and low-penetrance variants in the *ESR1* region associated with BC have been reported [43–46]. For genetic variants in rs2228480 and rs2077647, we did not find the significant association with the increased risk of BC, which was consistent with the findings of GWAS [47–49]. Our meta-analysis found that for rs3798577 the associations were diversity among different ethnic populations, but GWAS studies do not replicated it, the possible reason is that it not meet the standard of a significant result in GWAS studies. So a large population-based study needed be conducted to verify the ethnic diversity on the relationship between the genetic variant of rs3798577and BC risks.

For rs2077647:T>C, on the one hand, some studies [19, 23, 40] have shown that it has a protective effect against susceptibility to BC, but no functional implications of rs2077647 on the abundance of *ESR1* mRNA or mRNA expression were detected. Furthermore, another study [40] indicated that rs2077647 did not affect exonic splicing. On the other hand, although *ESR1* rs2077647:T>C is a silent coding polymorphism located in exon 1, it is unlikely to alter the protein encoded by *ESR1*. One research [50] indicated that one possible reasons for inter-population differences in estrogen- mediated diseases is the diversity of allele frequencies for the rs2077647 among the different ethnic populations, and the other possibility is the effects of some changes in the products of the ESR1 gene. However, the biological mechanisms underlying this phenomenon and the specific function of this SNP remain unclear.

The rs3798577:T>C polymorphism is located in the 3’ UTR of *ESR1*. Although the underlying biological mechanism and its functionality are not yet known, one plausible hypothesis is that rs3798577 polymorphisms might be major regulators of ER-α expression and might modify mRNA stability and *ESR1* gene expression.

The rs2228480:G>A polymorphism is a silent polymorphism located in exon 8 of *ESR1* and a synonymous variant. The functionality of this SNP is not yet known, but it seems to act as a regulator. Exon 8 is involved in the assembly of the C-terminal region of ER-α, which contributes to the regulation of reciprocal action between ER-α and other transcription factors [18]. Although rs2228480 does not alter amino acid sequences [16], rs2228480 has been suggested to modify the structure of mRNA, its splicing stability and the processes involved in its translation.

The present study had several strengths. Most importantly, it was the first meta-analysis conducted to evaluate the association between rs2077647 and BC risk. It was also the biggest and most recent meta-analysis of the association of rs2228480 and rs3798577 with BC risk, and it was more powerful than previous cohort and case-control studies. In addition, a subgroup analysis was conducted and demonstrated that the *ESR1* rs3798577:T>C polymorphism was associated with BC risk in a manner that depended on patient ethnicity.

However, some limitations of this meta-analysis must be addressed. First, the sample size was relatively small for stratified analyses and might not have provided sufficient power to estimate the associations. Second, the overall OR was based on individual unadjusted ORs, and some important confounding factors, such as age, sex, menopausal status, and BMI, must be adjusted for. Finally, although the funnel plots and Egger’s tests showed that publication bias did not affect our results, only studies published in English or Chinese were included, which produced selection bias at the start of our study.

In conclusion, our meta-analysis indicated that the *ESR1* rs3798577:T>C polymorphism might be a risk factor for BC in Asians and that the *ESR1* rs3798577:T>C polymorphism and *ESR1* rs2228480:A>G polymorphism had a large protective effect in Caucasians, while the *ESR1* rs2077647:T>C polymorphism was not associated with BC risk. However, the functions of these SNP gene variants in the development of BC and the full mechanisms underlying their effects are still unclear. In the future, more comprehensive and well-designed studies should be conducted to re-evaluate the associations of these three SNPs and other *ESR1* gene polymorphisms with BC risk.

## Supporting Information

S1 PRISMA Checklist PRISMA checklist. (DOCX)

S2 Checklist Meta-analysis of genetic association studies checklist. (DOCX)

S1 Fig pdf LD plots for the different populations. (PDF)

S1 Table List of excluded full-text articles. (XLSX)

S2 Table NOS-based quality assessment of the 20 eligible studies. (DOCX)

S3 Table Detailed genotype and allele frequency information. (XLSX)

S4 Table Detailed information for SNP rs2077647 in the four different models. (XLSX)

S5 Table Detailed information for SNP rs2228480 in the four different models. (XLSX)

S6 Table Detailed information for SNP rs3798577 in the four different models. (XLSX)

S7 Table Characteristics of the studies included in the meta-analysis of the three SNPs. (DOCX)

S1 Text Search strategies. (DOCX)

S2 Text Newcastle—Ottawa Quality Assessment Scale. (DOCX)
