# Correction: A Meta-Analysis of the Association between *ESR1* Genetic Variants and the Risk of Breast Cancer

**DOI:** 10.1371/journal.pone.0191579

**Published:** 2018-01-17

**Authors:** Taishun Li, Jun Zhao, Jiaying Yang, Xu Ma, Qiaoyun Dai, Hao Huang, Lina Wang, Pei Liu

The affiliation for the eighth author is incorrect. Pei Liu is not affiliated with #2 but with #1 Department of Epidemiology and Biostatistics, School of Public Health, Southeast University, Nanjing, China.
