# Supplementary material for: A Meta-Analysis of the Association between ESR1 Genetic Variants and the Risk of Breast Cancer
Source: PLoS One. 2016 Apr 12;11(4):e0153314. doi: 10.1371/journal.pone.0153314 (PMC4829239; doi:10.1371/journal.pone.0153314)
Supplement: S1 Fig — (PDF) [file pone.0153314.s003.pdf]

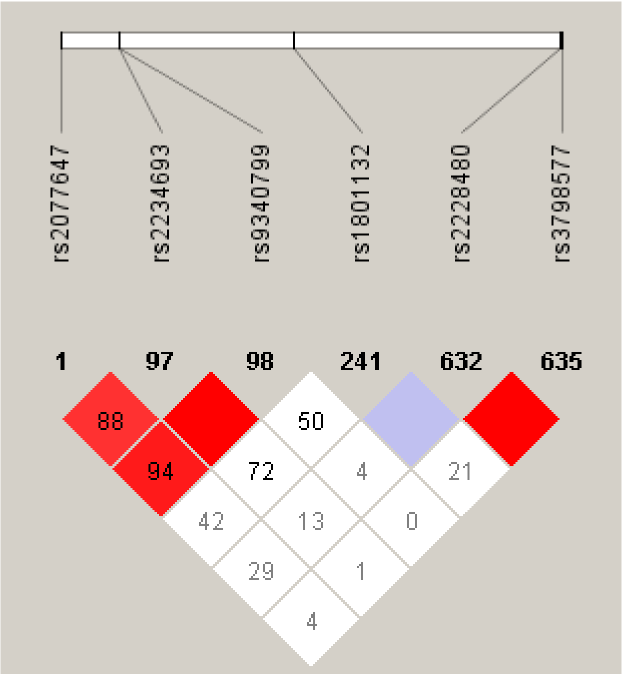

Fig 1. LD plot for the CEU(Utah residents with ancestry from northern and western Europe)

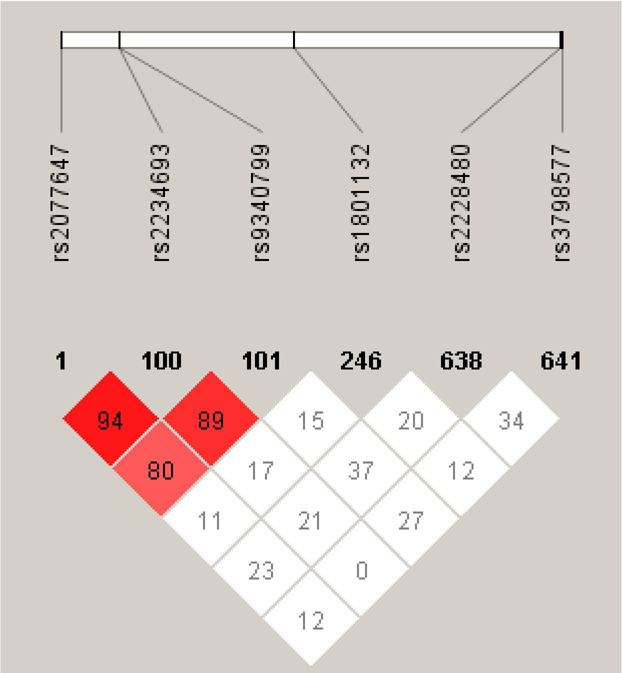

Fig 2. LD plot for the CHB(Han Chinese in Beijing, China)

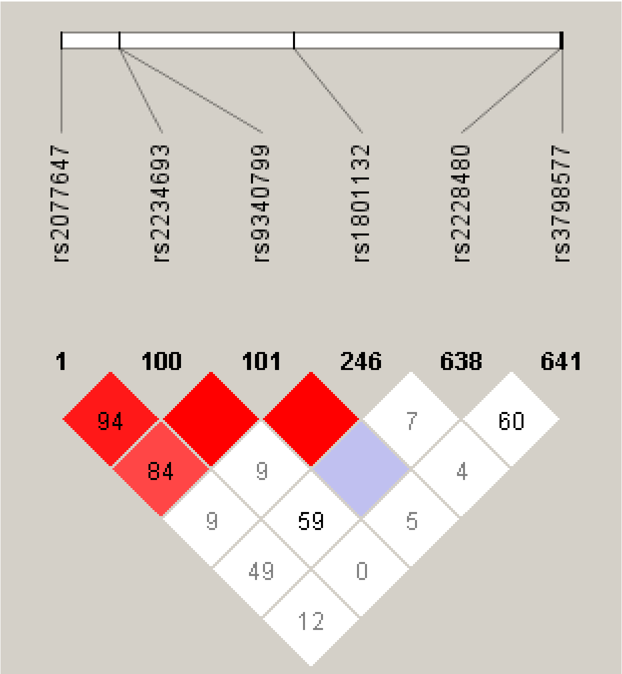

Fig 3. LD plot for the JPT(Japanese in Tokyo, Japan)

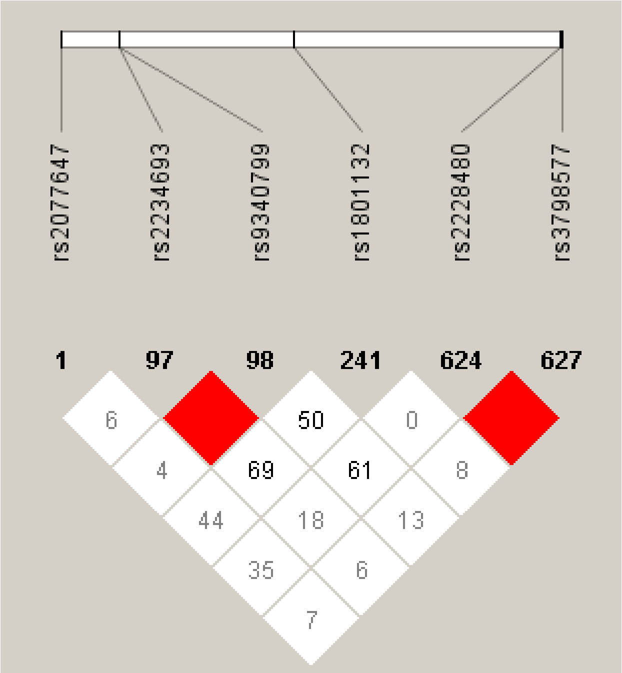

Fig 4. LD plot for the YRI(Yoruba in Ibadan, Nigeria)

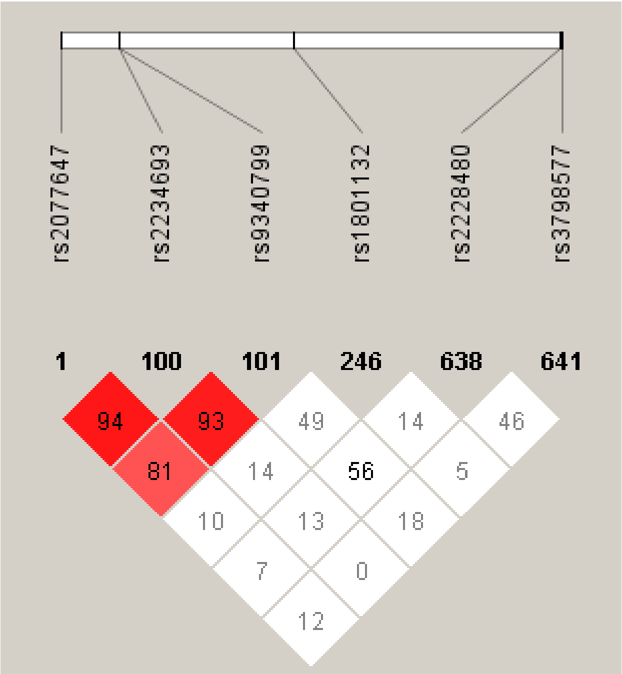

Fig 5. LD plot for the JPT and CHB
