# Supplementary material for: A Meta-Analysis of the Association between ESR1 Genetic Variants and the Risk of Breast Cancer
Source: PLoS One. 2016 Apr 12;11(4):e0153314. doi: 10.1371/journal.pone.0153314 (PMC4829239; doi:10.1371/journal.pone.0153314)
Supplement: S2 Table — (DOCX) [file pone.0153314.s005.docx]

**S 3 The results of quality assessment of the 20 eligible studies by using NOS**

| Frist Author | Published year | Study  method | Selection | Comparability | Exposure/Outcome | Quality  scores |
| --- | --- | --- | --- | --- | --- | --- |
| Son, B. H. | 2014 | CC | ★★★★ | ★ | ★★ | 7 |
| Jeon, S | 2010 | CC | ★★★ | ★★ | ★★ | 7 |
| Anghel, A | 2010 | CC | ★★★★ | ★ | ★★ | 7 |
| Yu,Jyh-Cherng | 2006 | CC | ★★★★ | ★ | ★★ | 7 |
| Wang, Y. R | 2014 | CC | ★★★★ | ★★ | ★★ | 8 |
| Gallicchio, L | 2006 | Cohort | ★★★★ | ★★ | ★★★ | 9 |
| Hsiao, W. C | 2004 | CC | ★★★★ | ★ | ★★ | 7 |
| Bosviel, Rémy | 2012 | CC | ★★★ | ★ | ★★ | 7 |
| Kallel, Imen | 2009 | CC | ★★★★ | ★★ | ★★ | 7 |
| Wang, J | 2013 | CC | ★★★★ | ★ | ★★★ | 8 |
| Tapper, William | 2008 | Cohort | ★★★★ | ★★ | ★★★ | 9 |
| Fernandez, L. P. | 2006 | CC | ★★★★ | ★ | ★★ | 7 |
| Nyante, SarahJ | 2015 | CC | ★★★★ | ★★ | ★★★ | 9 |
| Diergaarde, B | 2008 | CC | ★★★★ | ★★ | ★★ | 8 |
| Tse | 2006 | CC | ★★ | ★★ | ★★ | 6 |
| Xu, Yingchun | 2004 | CC | ★★★ | ★★ | ★★ | 7 |
| Zhang, L | 2009 | CC | ★★★★ | ★★ | ★★ | 8 |
| O'Brien, K. M | 2014 | CC | ★★★★ | ★★ | ★★ | 8 |
| SD Boone | 2013 | CC | ★★★ | ★★ | ★★ | 7 |
| Zhang,Lina | 2008 | CC | ★★★ | ★★ | ★★ | 7 |

*CC* case control study;The NOS identify ‘high’ quality choice with a ‘star’, a maximum of one ‘star’ for each item within the ‘Selection’ and ‘Exposure/Outcome’ categories; maximum of two ‘star’ for ‘Comparability’. ‘Exposure’ item were used to assess the cohort study, ‘Outcome’ item were used to assess the case-control study.
