# Supplementary material for: A Meta-Analysis of the Association between ESR1 Genetic Variants and the Risk of Breast Cancer
Source: PLoS One. 2016 Apr 12;11(4):e0153314. doi: 10.1371/journal.pone.0153314 (PMC4829239; doi:10.1371/journal.pone.0153314)
Supplement: S7 Table — (DOCX) [file pone.0153314.s010.docx]

**Table 1** Characteristics of studies included in meta-analysis of the three SNPs

| SNP | Author | Year | Cases | | | Controls | | | OR(95%CI) | Model | Adjusted  Factor | Power^c^ |
| --- | --- | --- | --- | --- | --- | --- | --- | --- | --- | --- | --- | --- |
|  |  |  | AA | Aa | aa | AA | Aa | aa |  |  |  |  |
| rs2228480 | Jeon, S | 2010 | 507 | 234 | 33 | 446 | 208 | 21 | 1.40(0.81-2.52) | AA vs. GG | Adjusted for age, education, age at the first full-term pregnancy, and family history of breast cancer | 0.326 |
|  | Anghel, A | 2010 | 61 | 41 | 1 | 62 | 21 | 1 | 1.01(0.06-16.61) | AA vs. GG | No | 0.019 |
|  | Yu,Jyh-Cherng | 2006 | 260 | 182 | 25 | 282 | 159 | 27 | 1.27(0.95-1.70) | (GA+AA) vs. GG | Adjusted for age, family history of breast cancer, full-term pregnancy, and body mass index | 0.956 |
|  | Wang, Y. R | 2014 | 682 | 340 | 40 | 633 | 387 | 49 | 0.84(0.72-0.98) | - | Adjusted for age, age at menarche and menopausal status | 0.407 |
|  | Gallicchio, L | 2006 | 58 | 20 | 2 | 841 | 358 | 41 | 1.42(0.34-6.01) | AA vs. GG | Adjusted for age | 0.156 |
|  | Hsiao, W. C | 2004 | 128 | 52 | 9 | 117 | 55 | 5 | - | - | - | - |
|  | Bosviel, Rémy | 2012 | 625 | 246 | 30 | 672 | 273 | 39 | - | - | - | - |
|  | Tapper, William^a^ | 2008 | 574 | 289 | 36 | 1763 | 1058 | 159 | 0.84(0.73-0.95) | A vs. G | NO | 0.462 |
|  | Wang, J^a^ | 2013 | 133 | 65 | 8 | 156 | 66 | 7 | 1.15(0.82-1.63) | A vs. G | NO | 0.090 |
|  | Kallel, Imen | 2009 | 101 | 36 | 5 | 147 | 76 | 17 | 2.33(0.83-6.53) | AA vs. GG | NO182 | 0.852 |
|  | Son, B. H^a^ | 2014 | 536 | 262 | 32 | 229 | 140 | 21 | 0.81(0.62-1.06) | (GA+AA) vs. GG | Adjusted for BMI, age at menarche, and age of first parturition | 0.390 |
| rs2077647 | Fernandez, L. P | 2006 | 184 | 238 | 113 | 152 | 260 | 127 | 0.74(0.53-1.02) | CC vs. TT | No | 0.697 |
|  | Nyante, SarahJ | 2015 | 534 | 986 | 452 | 461 | 913 | 399 | 0.99(0.81-1.20) | CC vs. TT | Adjusted for age, self-identified race, African ancestry et.al | 0.051 |
|  | Anghel, A | 2010 | 39 | 52 | 12 | 34 | 40 | 9 | 1.16(0.43-3.09) | CC vs. TT | No | 0.073 |
|  | Gallicchio, L | 2006 | 26 | 36 | 27 | 335 | 642 | 304 | 1.14(0.65-1.99) | CC vs. TT | Adjusted for age | 0.094 |
|  | Hsiao, W. C | 2004 | 91 | 75 | 23 | 69 | 73 | 35 | - | - | - | - |
|  | Diergaarde, B | 2008 | 86 | 148 | 90 | 176 | 291 | 184 | 1.00(0.80-1.40) | (CT+CC) vs. TT | NO | 0.050 |
|  | Tse | 2006 | 133 | 165 | 38 | 109 | 148 | 55 | 0.58(0.66-0.94) | CC vs. TT | NO | 0.881 |
|  | Xu, Yingchun^b^ | 2004 | 81 | 90 | 22 | 35 | 26 | 71 | - | - | - | - |
|  | Wang, J^a^ | 2013 | 68 | 101 | 37 | 75 | 113 | 42 | 0.99(0.76-1.30) | C vs. T | NO | 0.050 |
|  | O'Brien, K. M^a^ | 2014 | 315 | 630 | 315 | 473 | 908 | 436 | - | - | - | - |
|  | Son, B. H^a^ | 2014 | 318 | 392 | 120 | 172 | 174 | 44 | 1.37(1.05-1.79) | (GA+AA) vs. GG | Adjusted for BMI, age at menarche, and age of first parturition | 0.767 |
| rs3798577 | Zhang, L | 2009 | 114 | 131 | 55 | 121 | 183 | 86 | 1.37(0.84-2.23) | TT vs. CC | ORs were adjusted for age, education, BMI | 0.533 |
|  | Nyante, SarahJ | 2015 | 577 | 977 | 417 | 527 | 851 | 398 | 0.94(0.78-1.14) | CC vs. TT | Adjusted for age, self-identified race, African ancestry et.al | 0.080 |
|  | Wang, Y. R | 2014 | 336 | 527 | 196 | 305 | 541 | 226 | 0.90(0.79-1.02) | A vs. G | Adjusted for age, age at menarche and menopausal status | 0.200 |
|  | Fernandez, L. P | 2006 | 155 | 260 | 114 | 169 | 259 | 119 | 1.04(0.75-1.46) | CC vs. TT | NO | 0.061 |
|  | Anghel, A | 2010 | 8 | 59 | 36 | 30 | 41 | 18 | 7.50(2.86-19.65) | CC vs. TT | No | 0.999 |
|  | Tapper, William^a^ | 2008 | 226 | 450 | 223 | 833 | 1485 | 662 | 1.11(1.00-1.24) | C vs. T | NO | 0.279 |
|  | Wang, J^a^ | 2013 | 75 | 99 | 32 | 79 | 112 | 39 | 0.93(0.71-1.22) | C vs. T | No | 0.065 |
|  | SD Boone^a^ | 2013 | 185 | 341 | 157 | 232 | 345 | 128 | 1.36(1.04-1.76) | (CT+TT) vs. CC | NO | 0.798 |
|  | O'Brien, K. M^a^ | 2014 | 341 | 629 | 290 | 511 | 905 | 401 | - | - | - | - |
|  | Zhang,Lina^b^ | 2008 | 114 | 131 | 55 | 121 | 183 | 86 | - | - | - | - |
|  | Son, B. H^a^ | 2014 | 338 | 383 | 109 | 130 | 190 | 70 | 0.76(0.58-1.00) | (CT+TT) vs. CC | Adjusted for BMI, age at menarche, and age of first parturition | 0.572 |

*HWE* Hardy-Weinberg equilibrium, *MAF* minor allele frequency, *A* major allele, *a* minor allele, *OR* odd ratio, *CI* confidence interval

^a^ Genotype frequencies data were not suppled and calculated according to raw data.

^b^ Study published in Chinese language

^c^ Power was calculated by the PASS 11.0 software with MAF in controls as the frequency of risk factor and OR value in respective model as the relative risk.
