# Supplementary material for: A Meta-Analysis of the Association between ESR1 Genetic Variants and the Risk of Breast Cancer
Source: PLoS One. 2016 Apr 12;11(4):e0153314. doi: 10.1371/journal.pone.0153314 (PMC4829239; doi:10.1371/journal.pone.0153314)
Supplement: S1 Text — (DOCX) [file pone.0153314.s011.docx]

**Literature Search**

**The full details of the databases searched to identify the studies.**

The PUBMED, Web of Science, EBSCO and EMBASE electronic databases were explored using broad search strategies to identify all study evaluating the association between the ESR1 genetic variants and risks of breast cancer. All searches were run from the earliest date available (1966 for PUBMED, 1970 for Web of Science, 1988 for EMBASE) until October 2015. All indexed journals were included and retrieved. In addition, Google Scholar was also used to search relevant study. In order to search and include all potential studies, we applied various combinations of the following medical subject headings and key words in order to hold high sensitivity:

**Search Items:** ( “genetic polymorphism” or “single nucleotide polymorphism” or “SNP” or “gene mutation” ) and (“breast cancer” or “breast neoplasm” or “carcinogenesis” or “breast carcinoma” or “breast tumor” or “BC” or “mammary cancer”) and (“ESR1” or “Estrogen receptor α” or “ER alpha” or “Estrogen receptor alpha” or “ERα”).

All the general medical journals subscribed by our university has been searched. We therefore hand searched BMJ, AMA, Elsevier, Science Online, Springer and Nature from library. We routinely reviewed the content of those medical journals for the year up until the end of October 2015.Chinese papers were selected by searching WanFang Data, Chongqing VIP ( CQVIP), China National Knowledge Infrastructure(CNKI) databases using the same search terms. The references of the eligible articles were also inspected to find other potential studies. As a search limit, only studies published in English or Chinese were included.
